# Geschlechtsspezifische Unterschiede in Diagnostik und Therapie entzündlich-rheumatischer Erkrankungen

**DOI:** 10.1007/s00108-023-01484-3

**Published:** 2023-03-06

**Authors:** Katinka Albrecht, Anja Strangfeld

**Affiliations:** 1 Programmbereich Epidemiologie und Versorgungsforschung, Deutsches Rheuma-Forschungszentrum Berlin, Charitéplatz 1, 10117 Berlin, Deutschland; 2 Medizinische Klinik mit Schwerpunkt Rheumatologie und Klinische Immunologie, Charité – Universitätsmedizin Berlin, Berlin, Deutschland

**Keywords:** Rheumatoide Arthritis, Spondyloarthritis, Kollagenosen, Geschlechtsunterschiede, Antirheumatika, Arthritis, rheumatoid, Spondyloarthritis, Connective tissue diseases, Sex characteristics, Antirheumatic agents

## Abstract

**Hintergrund:**

Geschlechtsunterschiede in Diagnostik und Therapie verschiedenster Erkrankungen werden zunehmend erforscht mit dem Ziel, Therapiestrategien zu optimieren und den individuellen Behandlungserfolg zu verbessern.

**Methodik:**

In dieser Arbeit wird die bestehende Literatur zu geschlechtsspezifischen Unterschieden bei entzündlich-rheumatischen Erkrankungen zusammengefasst.

**Ergebnisse:**

Viele, aber nicht alle entzündlich-rheumatischen Erkrankungen treten häufiger bei Frauen als bei Männern auf. Frauen haben oft eine längere Beschwerdedauer bis zur Diagnosestellung als Männer, was durch unterschiedliche klinische und radiologische Erscheinungsbilder verursacht sein kann. Frauen haben krankheitsübergreifend häufiger niedrigere Remissions- und Therapieansprechraten in Bezug auf die antirheumatische Medikation als Männer, auch Therapieabbrüche sind bei Frauen häufiger als bei Männern. Ob Frauen vermehrt Anti-drug-Antikörper gegen biologische Antirheumatika entwickeln, ist noch unklar. Bei Januskinaseinhibitoren gibt es bislang keine Hinweise auf ein unterschiedliches Therapieansprechen.

**Schlussfolgerung:**

Ob auch in der Rheumatologie individuelle Dosierungsschemata und geschlechtsangepasste Therapieziele nötig sind, kann aus der bisherigen Evidenz nicht abgeleitet werden.

## Abstract (en)

**Background:**

Gender differences in the diagnosis and treatment of various diseases are increasingly being researched with the aim of optimizing treatment strategies and improving individual treatment success.

**Methods:**

This paper summarizes the existing literature for gender differences in inflammatory rheumatic diseases.

**Results:**

Many, but not all, inflammatory rheumatic diseases occur more frequently in women than in men. Women more often have a longer duration of symptoms until diagnosis than men, which may be due to different clinical and radiological presentations. Across diseases, women more often have lower remission and treatment response rates to antirheumatic medication compared to men. Discontinuation rates are also higher in women than in men. Whether women are more likely to develop anti-drug antibodies to biologic disease-modifying antirheumatic drugs is still unclear. For Janus kinase inhibitors, there is no evidence of differential treatment response to date.

**Conclusion:**

Whether individual dosing regimens and gender-adapted remission criteria are also required in rheumatology cannot be deduced from the evidence available to date.

Biologische und soziokulturelle Unterschiede zwischen Frauen und Männern können sowohl die Ausprägung entzündlich-rheumatischer Erkrankungen als auch deren Versorgung beeinflussen. Neben genetischen Merkmalen und Geschlechtshormonen unterscheiden sich auch Organgrößen und -funktionen, Körperbeschaffenheit und physiologische Prozesse zwischen den Geschlechtern. Dies betrifft beispielsweise die Schmerzverarbeitung oder Immunität, sodass bereits rein biologische Prozesse zu vielfältigen Unterschieden im Vorkommen, in der Symptomatik und auch im Therapieansprechen bei entzündlich-rheumatischen Erkrankungen führen können.

Es besteht großes Interesse, eine gendergerechte Forschung in der Rheumatologie zu etablieren

Soziokulturelle Unterschiede in der Wahrnehmung von Schmerzen, im Gesundheitsverhalten oder in den Auswirkungen krankheitsbedingter Einschränkungen auf die soziale und berufliche Teilhabe sind noch wenig erforscht. Die heutigen Therapieziele bei rheumatischen Erkrankungen umfassen aber nicht nur das Erreichen einer Remission im Sinne einer Normalisierung der klinischen Symptome und der serologischen Entzündungsmarker sowie die Vermeidung bzw. das Aufhalten struktureller Schäden, sondern sie beinhalten auch die Funktionsverbesserung und den Erhalt von Lebensqualität, inklusive der Möglichkeit, am sozialen und beruflichen Leben teilzunehmen [1]. Diese Aspekte sind gendersensibel. Durch den Wirkmechanismus eines Medikaments können unterschiedliche Merkmale der Erkrankung, beispielsweise Funktion, Fatigue, Schmerz oder Beweglichkeit, besonders wirksam adressiert werden. Dieser Effekt sollte genutzt werden, um geschlechtsspezifische Unterschiede der Krankheitsausprägung durch eine gezielte Therapieauswahl zu adressieren. Es gibt national wie international großes Interesse, eine gendergerechte Forschung in der Rheumatologie zu etablieren.

In diesem Beitrag soll auf die geschlechtsspezifischen Unterschiede in Diagnostik und Therapie entzündlich-rheumatischer Erkrankungen eingegangen werden, die vor allem für die internistische Versorgung relevant sind. Wir beziehen uns auf publizierte Arbeiten sowie auf aktuelle Daten der Kerndokumentation der Rheumazentren, einer seit 1993 jährlich durchgeführten Erhebung der Versorgungssituation und Krankheitslast von Patient:innen mit entzündlich-rheumatischen Erkrankungen.

## Geschlechterverteilung rheumatischer Erkrankungen

**Figure 1:**
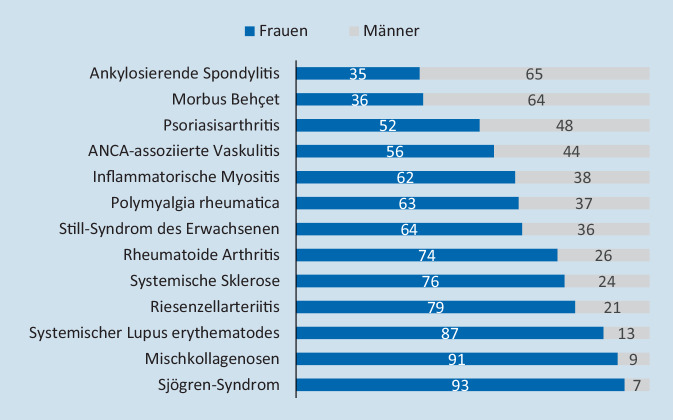

Bei den meisten, aber nicht bei allen rheumatischen Erkrankungen überwiegt – mit unterschiedlicher Ausprägung – der Anteil an Frauen (Abb. 1). Vor allem Kollagenosen wie das Sjögren-Syndrom, der systemische Lupus erythematodes oder die systemische Sklerose, aber auch die rheumatoide Arthritis (RA) entstehen häufiger bei Frauen, während die Geschlechterverteilung etwa bei Psoriasisarthritis ausgewogen ist. Durch die Erweiterung der Klassifikationskriterien für axiale Spondyloarthritiden auf Formen ohne sichtbare radiologische Veränderungen (sogenannte nichtradiologische Spondyloarthritis) hat sich das Geschlechterverhältnis bei der Gesamtgruppe der axialen Spondyloarthritis zwischen Frauen und Männern angeglichen, während sich die durch radiologische Veränderungen definierte ankylosierende Spondylitis häufiger bei Männern manifestiert [2]. Auch der Morbus Behçet tritt häufiger bei Männern auf.

## Unterschiedliche Dauer bis zur Diagnosestellung

Bereits bei Diagnosestellung unterscheidet sich die Symptomdauer bei Frauen und Männern, insbesondere bei entzündlichen Wirbelsäulenerkrankungen. Frauen mit axialer Spondyloarthritis und Psoriasisarthritis haben eine längere Beschwerdedauer, bis die Diagnose gestellt wird [4, 5]. Dies lässt sich nicht allein durch unterschiedliche Ausprägung der Symptome erklären [6]. Eine weitere mögliche Ursache sind Unterschiede in der klinischen Präsentation: Männer mit einer Spondyloarthritis weisen häufiger und früher radiologische Veränderungen sowie erhöhte serologische Entzündungsmarker auf, während bei Frauen vermehrt periphere Gelenke betroffen sind [2]. Dies kann in der Frühphase der Erkrankung zu Verwechslungen mit primär peripher auftretenden Gelenkerkrankungen führen. In einer spanischen Studie mit Patient:inneninterviews berichteten weibliche und männliche Patient:innen vergleichbare Symptome. Dennoch dokumentierten die behandelnden Ärzt:innen deutlich häufiger Rückenschmerzen bei Männern (82 % vs. 44 %), periphere Symptome hingegen häufiger bei Frauen (56 % vs. 18 %; [7]). Eine Beeinflussung durch ärztlich erwartete geschlechtsdifferente Symptome ist eine mögliche Erklärung.

Im Falle der Psoriasisarthritis tritt bei Männern häufiger eine für dieses Krankheitsbild typische Oligoarthritis auf, also ein Befall von nur einem oder wenigen Gelenken, während Frauen häufiger eine Polyarthritis aufweisen [5, 8], die von einer RA schwieriger zu differenzieren ist. Männer haben auch häufiger eine schwere Form der Psoriasis [5, 9], wodurch die Diagnosestellung erleichtert ist.

Obwohl Frauen häufiger an einer systemischen Sklerose erkranken, zeigen Daten aus der Kerndokumentation, dass es bei Frauen durchschnittlich ein Jahr länger bis zur Diagnosestellung dauert als bei Männern [10]. In den frühen Stadien der systemischen Sklerose liegt bei Männern nicht nur häufiger eine aktive Erkrankung vor als bei Frauen, sondern auch spezifische Anti-Scl-70-Antikörper, erhöhte Akute-Phase-Marker und eine muskuläre und pulmonale Beteiligung [11], sodass diese diagnostischen Marker bei Männern mutmaßlich früher zu einer Diagnose führen. Selbst beim systemischen Lupus erythematodes, der in einem Verhältnis von 8–9:1 überwiegend Frauen betrifft, wurde die Diagnose in einer spanischen Kohorte bei Männern schneller gestellt. Eine mögliche Erklärung ist die bei Männern oftmals vorliegende schwere Organbeteiligung – in dieser Studie hatten die männlichen Patienten häufiger kardiovaskuläre Komorbiditäten, Serositiden, Adenopathien, Splenomegalie, Nierenbeteiligung, Krampfanfälle, Thrombosen und ein positives Lupusantikoagulans als die Patientinnen [12].

## Ungleichheiten in der Inanspruchnahme der rheumatologischen Versorgung

Die spätere Diagnosestellung bei Patientinnen ist umso bemerkenswerter, als das Gesundheitsverhalten von Frauen eher zu einer rechtzeitigeren Diagnose führen müsste. In einer kanadischen Kohortenstudie konsultierten Patientinnen in den drei Jahren vor Diagnose einer RA, Psoriasisarthritis oder ankylosierenden Spondylitis häufiger eine Rheumatolog:in und hatten auch häufiger labor- und bildgebende Untersuchungen. Auch nach der Diagnose blieben die Patientinnen häufiger in der rheumatologischen Versorgung als die männlichen Patienten [13, 14]. In einer weiteren kanadischen Analyse wurde offenbar, dass männliche Hausärzte später eine rheumatologische Überweisung veranlassten als ihre Kolleginnen [15]. Folglich kann auch das ärztliche Geschlecht zu Unterschieden in der Versorgung beitragen.

## Unterschiede in Organmanifestation und Komorbiditäten

Ausmaß und Stadium von Organmanifestationen sind bei vielen entzündlich-rheumatischen Erkrankungen maßgeblich für das Fortschreiten der Erkrankung und das Risiko des Versterbens der Patient:innen. Die autoimmunassoziierte interstitielle Lungenerkrankung („interstitial lung disease“ [ILD]) ist eine Organmanifestation vieler rheumatischer Systemerkrankungen [16], die oft mit einer schlechten Prognose einhergeht. Auch wenn Frauen insbesondere in der hormonaktiven Lebensphase häufiger an einer systemischen Sklerose erkranken, haben Männer insgesamt ein erhöhtes Risiko für eine assoziierte ILD. Die systemische Sklerose verläuft bei Männern oftmals stärker progredient als bei Frauen (mit höherer Krankheitsaktivität, häufigerem Auftreten digitaler Ulzerationen und pulmonaler Hypertonie) und geht mit einem 2fach erhöhten Risiko einher, daran zu versterben [17]. In einer Post-hoc-Auswertung zweier randomisierter klinischer Studien verschlechterten sich die Vitalkapazität und der radiologische Befund bei Männern unter Therapie mit Cyclophosphamid oder Mycophenolat deutlicher als bei Frauen, einhergehend mit einem erhöhten Mortalitätsrisiko. In der bronchoalveolären Lavage hatten Männer erhöhte Konzentrationen an profibrotischen Mediatoren, während Frauen höhere Konzentrationen an proinflammatorischen Mediatoren aufwiesen [18].

Frauen und Männer unterscheiden sich in ihrem Spektrum an Begleiterkrankungen

**Figure 2:**
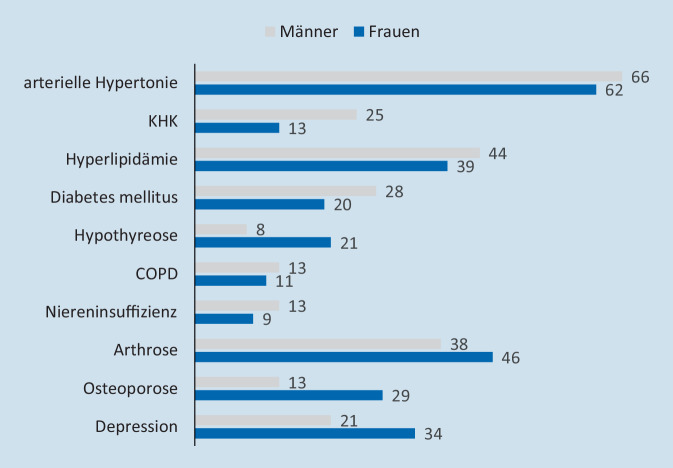

Komorbiditäten beeinflussen die Therapieauswahl, das Therapieansprechen und den weiteren Verlauf entzündlich-rheumatischer Erkrankungen. Frauen und Männer unterscheiden sich in ihrem Spektrum an Begleiterkrankungen. Während Frauen mit RA häufiger Arthrosen, Osteoporose, Depression und Schilddrüsenerkrankungen haben, sind es bei Männer eher kardiovaskuläre Erkrankungen, Diabetes, Gicht und Niereninsuffizienz (Abb. 2). Übergewicht tritt zunehmend bei Frauen auf [5, 19].

Herz-Kreislauf-Erkrankungen sind die Haupttodesursache von Patient:innen mit entzündlich-rheumatischen Erkrankungen. Auch hier gibt es geschlechtsspezifische Unterschiede. Im Biologikaregister RABBIT („Rheumatoide Arthritis: Beobachtung der Biologika-Therapie“) hatten Männer im Vergleich zu Frauen bei RA ein höheres Risiko sowohl für die Entwicklung einer Herzinsuffizienz als auch für einen schlechteren Verlauf und eine höhere Mortalität einer bestehenden Herzinsuffizienz [21, 22]. Echokardiographische Untersuchungen in einer italienischen RA-Kohorte zeigen, dass bei Frauen häufiger als bei Männern eine linksventrikuläre Hypertrophie bestand, die mit erhöhten systemischen Entzündungsmarkern assoziiert war [23]. Das kardiovaskuläre Risiko wird bei Frauen vermutlich unterschätzt – in einer niederländischen Kohortenstudie wurde bei 36 % der weiblichen, aber nur bei 10 % der männlichen RA-Patienten mit einem kardialen Ereignis das kardiovaskuläre Risiko zuvor als niedrig eingeschätzt [24]. In der aktuellen Diskussion um das Risiko kardiovaskulärer Ereignisse und venöser Thrombosen unter Januskinase(JAK)-Inhibitoren im Vergleich zu Tumor-Nekrose-Faktor(TNF)-Inhibitoren gibt es bisher keinen Hinweis auf geschlechtsspezifische Risiken [25].

## Unterschiedliche Krankheitsbelastungen

Auch wenn die Psoriasisarthritis gleich häufig Frauen und Männer betrifft, ist die subjektive Krankheitsbelastung bei Frauen stärker ausgeprägt als bei Männern [9]. Sie geben eine höhere Krankheitsaktivität, mehr schmerzhafte Gelenke, intensivere Schmerzen, häufiger Fatigue und stärkere Funktionseinschränkungen an als Männer [5, 9]. Größere Einschränkungen zeigen sich auch in der Lebensqualität und beruflichen Teilhabe [26]. Die Wirkung der Sexualhormone auf das Schmerzempfinden sowie Unterschiede in der zentralen Schmerzverarbeitung werden als Faktoren mit Einfluss auf die von den Patient:innen angegebenen Schmerzen diskutiert [9]. Hierzu passen Daten aus der Früharthritiskohorte CAPEA: Frauen, die aktuell oder anamnestisch orale Kontrazeptiva eingenommen hatten, gaben deutlich weniger Schmerzen und Fatigue sowie geringere Funktionseinschränkungen an als Frauen ohne orale Kontrazeption [27].

Etwa die Hälfte und mehr weibliche als männliche Patient:innen mit RA, ankylosierender Spondylitis oder Psoriasisarthritis schätzen ihre Krankheitsaktivität höher ein als die behandelnden Rheumatolog:innen. Die unterschiedliche Beurteilung war aber bei weiblichen und männlichen Rheumatolog:innen identisch [28].

## Wirksamkeit der Therapie mit „disease-modifying antirheumatic drugs“

Daten einer niederländischen Früh-Psoriasisarthritis-Kohorte zeigen, dass bei ähnlicher initialer Therapiestrategie Frauen kürzer als Männer mit Methotrexat (196 vs. 306 Tage) behandelt wurden und eine niedrigere kumulative Dosis (543 mg vs. 757 mg) erhielten. Darüber hinaus wurden biologische „disease-modifying antirheumatic drugs“ (DMARD) bei Männern früher eingesetzt als bei Frauen. Nach einem Jahr Therapie erreichten die Frauen in der Kohorte deutlich seltener als die Männer das Therapieziel einer minimalen Krankheitsaktivität (36 % vs. 58 %) bzw. einer Remission (11 % vs. 28 %; [5]). In einer randomisierten Studie zu Psoriasisarthritis sprachen männliche Probanden auf den TNF-Inhibitor Etanercept besser an als weibliche, unabhängig von den verwendeten Scores [29]. Auch für die ankylosierende Spondylitis zeigt eine Auswertung des spanischen BIOBADASER-Registers, dass Frauen schlechter auf den ersten TNF-Inhibitor ansprechen als Männer [30]. Bei den JAK-Inhibitoren sprechen erste Untersuchungen dafür, dass es keinen geschlechtsspezifischen Unterschied im Therapieansprechen gibt [31].

Auch Komorbiditäten können das Therapieansprechen geschlechtsspezifisch beeinflussen. Ergebnisse aus dem Biologikaregister RABBIT zeigen, dass Übergewicht bei Frauen noch mehr als bei Männern die Wirksamkeit zytokingerichteter Therapeutika wie TNF-Inhibitoren und Tocilizumab reduziert [32].

## Sicherheitsaspekte

Über alle Arzneimittelklassen hinweg haben Frauen ein fast doppelt so hohes Risiko unerwünschter Wirkungen wie Männer [33]. Unter der DMARD-Therapie im ersten Erkrankungsjahr gaben Frauen aus der niederländischen Früh-Psoriasisarthritis-Kohorte häufiger Nebenwirkungen an als Männer (58 % vs. 42 %; [5]). In einer Metaanalyse war auch bei RA weibliches Geschlecht ein Risikofaktor für einen früheren Biologikatherapieabbruch [34]. Unterschiede in der Pharmakokinetik und im Körpergewicht können dazu beitragen. So nehmen das Verteilungsvolumen und die Clearance monoklonaler Antikörper mit der Körpergröße zu [31]. Bei Patient:innen mit RA wurde für Adalimumab [35] und für Rituximab [36] eine höhere Clearance bei Männern als bei Frauen nachgewiesen. Eine individuelle Dosierung der Therapie mit monoklonalen Antikörpern erscheint daher sinnvoll, doch entsprechende Studien fehlen. Die Entwicklung von Anti-drug-Antikörpern ist ein Grund für den sekundären Wirkverlust biologischer DMARD-Therapien. Publizierte Ergebnisse zu Geschlechterunterschieden widersprechen sich jedoch: In der randomisierten SWEFOT-Studie entwickelten Frauen mit RA unter einer Kombinationstherapie mit Rituximab und Methotrexat häufiger Anti-drug-Antikörper als Männer [37], während dies in einer Kohortenstudie mit Patient:innen mit entzündlicher Darmerkrankung unter Rituximab vermehrt bei Männern beobachtet wurde. Unter Adalimumab gab es dagegen keine geschlechtsspezifischen Unterschiede [38].

Frauen haben unter Allopurinol eine deutlich schlechtere Compliance und höhere Abbruchraten

Bei Allopurinol ist bekannt, dass Frauen ein 2,5fach höheres Risiko haben, schwere kutane Arzneimittelreaktionen zu entwickeln [39]. Darüber hinaus haben Frauen auch eine deutlich schlechtere Compliance und höhere Abbruchraten unter dieser Therapie [40]. Das Therapieansprechen auf Allopurinol oder Benzbromaron unterscheidet sich bei Frauen und Männern jedoch nicht [41].

Bei Frauen treten unter Nintedanib zur Behandlung der interstitiellen Lungenerkrankung vermehrt unerwünschte Ereignisse wie Übelkeit, Erbrechen und Transaminasenanstieg auf. Diese führen in der Folge häufiger zu einer Dosisreduktion bzw. Therapieunterbrechung, als es bei Männern der Fall ist [42]. Dahingegen haben Männer mit prognostisch ungünstiger systemischer Sklerose ein höheres Risiko als Frauen, in der Folge einer autologen Stammzelltransplantation zu versterben [43].

## Therapieanpassung bei Frauen und Männern mit Kinderwunsch

Sowohl Frauen als auch Männer mit entzündlich-rheumatischen Erkrankungen und Kinderwunsch sollten die Schwangerschaft planen und eine rheumatologische Beratung zu den möglichen Auswirkungen der Erkrankung, aber auch der antirheumatischen Medikation auf Fertilität und Schwangerschaftsverlauf erhalten.

Für Frauen mit Kinderwunsch gilt: Teratogene Antirheumatika wie Methotrexat, Mycophenolat und Cyclophosphamid müssen etwa drei Monate vor einer geplanten Konzeption abgesetzt werden [44]. Auch eine Leflunomidtherapie muss beendet und gegebenenfalls medikamentös ausgewaschen werden. Die Fortführung einer Therapie mit TNF-Inhibitoren ist möglich und wird je nach individueller Situation zumindest für das erste und zweite Trimester empfohlen, um die Erkrankung inaktiv zu halten [44]. Rituximab und Belimumab können bis zur Konzeption appliziert werden, das Vorgehen sollte individuell abgestimmt werden. Therapien mit Hydroxychloroquin, Sulfasalazin, Azathioprin, Ciclosporin, nichtselektiven nichtsteroidalen Antirheumatika und niedrig dosierten Glukokortikoiden können in der Schwangerschaft und Stillzeit eingesetzt werden [44]. Für Apremilast, JAK-Inhibitoren und neuere Biologika ist die Datenlage unzureichend, sodass bislang empfohlen wird, sie in der Schwangerschaft zu vermeiden.

Für Männer mit Kinderwunsch gilt: Cyclophosphamid kann auch bei Männern die Fruchtbarkeit beeinträchtigen und muss drei Monate vor Konzeption abgesetzt werden. Für weitere Antirheumatika gibt es einschränkende Empfehlungen zur Fortsetzung [45], die individuell besprochen werden sollten. So kann beispielsweise die Therapie mit TNF-Inhibitoren fortgeführt werden. Sulfasalazin kann die Bildung von Samenfäden und somit die Fertilität beeinträchtigen.

## Fazit für die Praxis

- In der Rheumatologie sind geschlechtsspezifische Unterschiede im Vorkommen entzündlich-rheumatischer Erkrankungen und hinsichtlich der Zeitdauer bis zur Diagnosestellung seit vielen Jahren bekannt.
- Zu Unterschieden im Therapieansprechen, bei Remissionsraten und in patientenberichteten Outcomes gibt es für einige rheumatische Krankheitsbilder bereits Evidenz.
- Geschlechtsspezifische Einflüsse auf Wirksamkeit und Nebenwirkungsprofil antirheumatischer Medikamente rücken gerade erst in den Fokus wissenschaftlicher Studien.
- Bislang gibt es keine Therapieempfehlungen oder Grenzwerte für Krankheitsaktivitätsscores, die das Geschlecht berücksichtigen.
- Ob auch in der Rheumatologie individuelle Dosierungsschemata und angepasste Outcomeparameter nötig sind, die das Geschlecht berücksichtigen, kann aus der derzeitigen Evidenz nicht abgeleitet werden. Die Genderforschung in der Kardiologie legt aber nahe, dass durch angepasste Therapiestrategien Überdosierungen und Therapieabbrüche reduziert werden können.
